# Reliability and Confirmatory Factor Analysis (CFA) of a Paper- Versus App-Administered Resilience Scale in Scottish Youths: Comparative Study

**DOI:** 10.2196/11055

**Published:** 2021-12-07

**Authors:** Julienne Mcgeough, Thomas Gallagher-Mitchell, Dan Philip Andrew Clark, Neil Harrison

**Affiliations:** 1 Department of Psychology Liverpool Hope University Liverpool United Kingdom

**Keywords:** resilience, psychometrics, app administration, cyberpsychology

## Abstract

**Background:**

Adequately measuring resilience is important to support young people and children who may need to access resources through social work or educational settings. A widely accepted measure of youth resilience has been developed previously and has been shown to be suitable for vulnerable youth. While the measure is completed by the young person on paper, it has been designed to be worked through with a teacher or social worker in case further clarification is required. However, this method is time consuming and, when faced with large groups of pupils who need assessment, can be overwhelming for schools and practitioners. This study assesses app software with a built-in avatar that can guide young persons through the assessment and its interpretation.

**Objective:**

Our primary objective is to compare the reliability and psychometric properties of a mobile software app to a paper version of the Child and Youth Resilience measure (CYRM-28). Second, this study assesses the use of the CYRM-28 in a Scottish youth population (aged 11-18 years).

**Methods:**

Following focus groups and discussion with teachers, social workers, and young people, an avatar was developed by a software company and integrated into an android smartphone app designed to ask questions via the device’s inbuilt text-to-voice engine. In total, 714 students from 2 schools in North East Scotland completed either a paper version or app version of the CYRM-28. A cross-sectional design was used, and students completed their allocated version twice, with a 2-week period in between each testing. All participants could request clarification either from a guidance teacher (paper version) or from the in-built software glossary (app version).

**Results:**

Test and retest correlations showed that the app version performed better than the paper version of the questionnaire (paper version: *r*_303_=0.81; *P*<.001; 95% CI 0.77-0.85; app version: *r*_413_=0.84; *P*<.001; 95% CI 0.79-0.89). Fisher r to z transformation revealed a significant difference in the correlations (Z=–2.97, *P*<.01). Similarly, Cronbach α in both conditions was very high (app version: α=.92; paper version: α=.87), suggesting item redundancy. Ordinarily, this would lead to a possible removal of highly correlated items; however, our primary objective was to compare app delivery methods over a pen-and-paper mode and was hence beyond the scope of the study. Fisher r to z transformation revealed a significant difference in the correlations (Z=–3.69, *P*<.01). A confirmatory factor analysis supported the 3-factor solution (individual, relational, and contextual) and reported a good model fit (*χ*^2^_15_=27.6 [n=541], *P*=.24).

**Conclusions:**

ALEX, an avatar with an integrated voice guide, had higher reliability when measuring resilience than a paper version with teacher assistance. The CFA reports similar structure using the avatar when compared against the original validation.

## Introduction

### Resilience

Resilience has traditionally been conceptualized as an individual difference. For example, early research in the field showed that some children, even when exposed to a chaotic family life or early life stressors (eg, bereavement) displayed surprisingly healthy behaviors; for example, coping ability [1-3]. Indeed, a child with high levels of resilience will be able to overcome stressors to achieve a sense of well-being [4]. Furthermore, in a review, Panter-Brick and Leckman [5] established a pathway between childhood resilience and adult well-being. However, as work on resilience has progressed, it has become increasingly recognized that factors external to the child may also influence later personal and academic success [1-3]. Luthar, Lyman, and Crossman [6] categorized subfactors of resilience into three themes, namely “Attributes of the individual,” “Family influences,” and “Wider social environments.” Ungar [7,8] further expanded on these categories to develop a dynamic concept of resilience that places society at the center of a child’s ability to develop resilience and coping strategies. Ungar’s *ecological model of resilience* is culturally sensitive, and while it does accept that there are individual differences in coping, it argues that the environment surrounding the individual is crucial in providing appropriate resources. For example, while Ungar’s definition and subsequent measurement includes differential aspects of the ability to maintain friendships, it also measures whether the young persons have been provided with the tools to do so. Ungar [7-9] further suggests that resilience definitions should reflect both ontological and ecological variability and states the following:

In the context of exposure to significant adversity, resilience is both the capacity of individuals to navigate their way to the psychological, social, cultural, and physical resources that sustain their well-being, and their capacity individually and collectively to negotiate for these resources to be provided and experienced in culturally meaningful ways.

In Scotland (the setting for this study), pupils are currently supported via guidance teachers within the Getting It Right For Every Child (GIRFEC) framework set by the Government, and well-being is conceptualized within SHANARRI. SHANARRI has 8 indicators of well-being: Safe, Healthy, Active, Nurtured, Achieving, Respected, Responsible, and Included [10,11]. Guidance teachers lead the pastoral support for pupils of all ages, generally with approximately 200-250 pupils within their care, and with whom they will have Personal and Social learning classes each week, along with additional support if required [12]. It is within this setting that well-being, resilience, and SHANARRI are measured. While there is a positive perception among pupils and parents regarding the support offered by guidance teachers, this is not consistent with a large minority of parents who argue that the system does not support their child [13]. The challenge for schools across Scotland is the government-led initiative in which they are expected to assess the risks and vulnerability of each child [14]. Clearly, this should easier to accomplish with an app that can measure resilience and well-being easily while engaging each pupil. Furthermore, the system is under strain as funding decreases, with the education system reducing the number of guidance teachers [15,16].

### Psychometric Measurement Using Apps

Ungar and Liebenberg [17,18] developed a scale of resilience, which reflected this definition of resilience and was expressed in 3 factors (individual, relational, and contextual). Sample items are “I cooperate with people around me” (individual), and “my caregivers watch me closely” (relational). The questionnaire is designed to be used as a verbally administered questionnaire, conducted by a professional within the setting, with responses measured on a Likert scale from 1 to 7. However, this is time-consuming and difficult to administer on an individual basis to large groups of pupils requiring assessment. Further studies have changed verbal administration of the questionnaire to a more traditional paper-based version to widen participation [19]. However, this obviously loses the verbal aspect of the questionnaire, which, according to Ungar [9], increases participants’ understanding. Therefore, an alternative to personal administration with each child is to use software that allows questions to be read if the participant requires it.

This study seeks to address the issue of scalability while retaining the verbal aspect and reducing the need for competent reading skills. A further advantage is the benefit of software-based data collection, which, according to current research, reduces the chances of incorrect or missing input and therefore increases validity and reliability [20]. Furthermore, there is evidence that internal consistency and concurrent validity are retained when transitioning to an app-based questionnaire. Importantly, app-based scales have consistently been shown to have higher completion rates among studies included in a large-scale meta-analysis [21]. However, it cannot be assumed that transitioning from a paper version to an app version will automatically carry over psychometric properties, though there is growing evidence that the transfer to computer-based measures does not result in a loss of psychometric properties [22]. However, this is transference of psychometric properties is by no means universal; for example, when transferring pen-and-paper psychometric questionnaires, Booth-Kewley et al [23] found that a level of disinhibition crept in to measures regarding such topics as alcohol consumption and risky sexual behaviors. Therefore, it is still necessary to validate the development of a software-based app. It is of crucial importance that this is undertaken when the design of the app differs from the original scale administration format, as in this study where an avatar is used to deliver the items. Traditionally, data collection on the internet was designed to closely resemble that with paper questionnaires; however, recent studies have explored nonhuman interaction (Bot) with humans and their tendency to disclose, with provide evidence that self-disclosure increases with the use of nonhuman interviewees [24].

### This Study

Our affinity for smartphones has been explained by various theories ranging from Bowlby’s attachment theory, addiction-based models, and emotional needs theories [25-27]. Indeed, it has been suggested that even larger portable technology, such as laptops, can be seen to be an extension of our identity and selves, given that we store memories through photographs and access social media on them [28]. For this study, these identity processes and dynamics are identified as being drivers in the adolescent relationship with their technological companions, which may be seen as an extension of “self” [29]. Furthermore, adolescents have been described as a population that is difficult to reach for research purposes; therefore, a smartphone app such as the one tested in this study should increase usability [30]. It has been proposed that the interaction of the aforementioned dynamics will encourage honesty in this population and therefore increase the reliability of the questionnaire, as reported in other studies exploring issues of well-being in hard-to-reach populations [31]. “Avatar as a researcher” is an emerging concept, and previous studies have shown increased trust and openness, thus increasing the reliability and confidence in data when discussing sensitive topics [32]. Identification with avatars and robots occurs with both humanoid and nonhumanoid avatars. For example, even computer-driven triangle shapes are perceived to have intentionality [33,34]. Therefore, it is expected that this study will see improved reliability, increased completion rates, and similar psychometric properties retained following validity analysis, in the app-based delivery. Additionally, this study aims to validate the use of the CYRM-28 among a Scottish population.

## Methods

### App Development

Feedback on a number of avatar designs was gathered from 30 professionals, including social workers, educational psychologists, and teachers, at the 2015 *Pathways to Resilience* Conference. The outcome of the discussions was to avoid humanoid-like avatars of similar ages to the participants, and to opt for one that would be considered gender neutral. ALEX has facial elements that move (eyes and mouth), and uses the speech-to-text engines of the device that is running the app. ALEX moves and bounces in response to screen touches. Further focus groups with young people confirmed that ALEX was user-friendly, approachable, and liked by a wide range of ages of both sexes. Participants in the app group were asked to complete a usability questionnaire following the resilience questionnaire.

### Design

Recruitment was carried out in schools that agreed to take part in trials. Information sheets were sent to parents electronically and parents could access a website about the research and agree to participate via web-based surveys. A cross-sectional design was used, which aimed at comparing the performances of pen-and-paper to that of an app-based CYRM-28 scale [17]. Two schools included all of their pupils, and classes were randomly designated as either app versus paper with age groups represented in each group. All groups were presented with the scale twice, with a 2-week retest design. Data collection was completed in Personal and Social Education (PSE) classes, and took approximately 10 minutes for the majority of the students. This was preceded by a short explanation regarding the administration of the scale and a reminder of their ethical rights. A guidance teacher and a member of the data collection team were present during the session. As with the original CYRM-28, participants could request further information and clarification from the researcher regarding the item statements (paper version) or an in-built glossary that could be accessed when the pupil highlighted a word or phrase. All research took place during the second term of the academic year (January to March 2017). A third school took part in 1 app-based data collection during the Summer term (July 2017) under the same conditions as described above, but further participation was prevented owing to end-term examination. These data are included only in the CFA.

### Participants

The participants were 714 students from 2 North-East Scotland coeducational schools, aged 11-17 years (males: n=354, mean age 14.3 years, SD 2.42 years; females: n=360, mean age 14.6 years, SD 2.37 years). Areas in Scotland are divided into 5 broad groupings of deprivation (1=most deprived to 5=least deprived) and are reported with the Scottish Index of Multiple Deprivation [35]. School 1 (n=403) includes a high-income area, and the majority of pupils fall into bands 4 and 5 (relatively high socioeconomic status [SES] in accordance with the Government’s deprivation bands). School 2 (n=311) is in an urban setting classified as a high deprivation area (all pupils are classed as being in the top 2 levels of deprivation). The final school draws from a wide range of SES bands. All 3 schools are comprehensives and therefore mixed-ability schools with intakes of pupils aged 16-18 years. The schools used mixed-ability groups, and each of the schools have approximately similar numbers on the roll.

### Materials

The app version ran on Kindle Fires (HD), which were disconnected from the internet, and other software could not be accessed. The app presents the questions via the ALEX avatar. ALEX is gender-neutral and is displayed in diagram 1 below, along with a typical question. As with the paper version, the students were required to respond on a 1-7–point Likert scale (strongly disagree to strongly agree), yielding a possible data range of 28-196, with a higher score indicating stronger resilience. The app version has a computerized voice, which is able to read the question to the participant, and a glossary of available terms. These had been tested by adolescents who had trialed the software and had indicated where they thought help would be required. In the pen-and-paper version, help was given if requested by the participant at the time, and adults provided the same answers as given by the predetermined glossary. There were no reports of pupils asking questions outside of this set. The scale has previously been found to have good reliability scores (individual: α=.803; relational: α=.833; contextual: α=.794), and adequate validity after exploratory and confirmatory analyses [17]. The project received ethical approval from the Liverpool Hope University Ethics board (S040417 SFREC 001), and students were required to read a short participation information sheet or screen after a short verbal reminder of their right to withdraw from the research. Parents had provided informed consent to their children’s participation. Demographic information and data regarding the usability of the app were collected.

### Statistical Analysis

For demographic descriptive statistics, only results from time 1 were included. All data met parametric assumptions. Items in the app condition were grouped and calculated to form 3 factors in accordance with an a priori theory developed by Liebenberg and Ungar [18]. The first factor (individual) was composed of 11 items which were further conceptualized as personal skills, peer support, and social skills. The second factor of relationships with caregivers included 7 items divided into physical and psychological care. The final factor was labeled as contextual and had 3 subfactors (educational, spiritual, and cultural).

Data from 12 respondents were removed prior to a CFA, following identification as multivariate outliers using the Mahalanobis Distance (MD) method. AMOS 24 was used to complete the CFA using a Maximum Likelihood Model. Files have been archived on the Open Science Forum [36].

## Results

### Usability Results

In total, 262 of the pupils took part in the usability questionnaire. The majority of the participants rated the app as easy to very easy to use (87.4%), compared to those who rated it hard or very hard (4.4%). Additionally, users were positive about their experience regarding interaction with ALEX. However, participants were moderately negative with the voice that read the instructions, with 31% stating that it needed to be changed. They were also encouraged to leave comments regarding improvements; in this field, the most common suggestion was to include a game.

### Assessment Results

Descriptive statistics for resilience are reported in Table 1. These data show that males and females reported similar scores and suggest minor differences in resilience across schools. Resilience scores decreased with age, with the youngest pupils aged 11 years reporting higher levels (mean 113.05, SD 11.85) than those aged >16 years (mean 103.50, SD 15.10). Pearson correlation analysis indicated a significant relationship between age and resilience (*r*=0.81; *P*=.006; 95% CI 0.02-2.73).

**Table 1:** Summary of the scores for each sample.

		Sample size, n		Score, mean (SD)		95% CI	
		S1^a^	S2^b^	S1	S2	S1	S2
**Paper version**							
	Total	82	126	107.85 (13.66)	104.22 (11.64)	104.08-110.09	102.17-106.27
	Males	36	50	108.06 (12.93)	103.06 (10.52)	103.68-112.24	100.04-106.08
	Females	45	76	106.53 (14.41)	105.01 (12.38)	102.20-110.86	102.19-107.84
**App version**							
	Total	234	183	107.45 (13.71)	105.95 (13.33)	105.69-109.21	104.01-107.90
	Males	135	97	107.65 (13.69)	106.38 (14.00)	105.30-110.02	10354-109.22
	Females	99	84	107.57 (13.65)	105.90 (12.57)	104.84-110.29	103.17-108.63

^a^S1: school 1 (deprivation groups 4 and 5).

^b^S2: school 2 (deprivation groups 1 and 2).

There was no difference between the schools in terms of resilience (school 1: mean 107.24, SD 12.87; school 2: mean 105.79, SD 13.15; *t*_720_=1.38; *P*=.18). In the paper version, scores on the CYRM-28 ranged from 63 to 131 (mean 106.98, SD 13.51); however, in the app version, the equivalent results were 56-135 (mean 106.79, SD 13.62). An independent samples *t* test was conducted between the 2 conditions and reported no significant difference (*t*_720_=–0.632; *P*=.53; 95% CI –2.55 to 1.31).

### Psychometric Properties

Cronbach α in both conditions was very high (app: α=.92; paper: α=.87). Fisher r to z transformation revealed a significant difference in the correlations (Z=–3.69, *P*<.01). Test-retest results (Pearson correlation coefficients) were significant in both conditions, although the app version had higher reliability (paper version: *r*_303_=0.81; *P*<.001; 95% CI 0.77-0.85; app version: *r*_413_=0.84; *P*<.001; 95% CI 0.79-0.89). As SPSS was used to calculate the 95% CIs with a linear regression model, *z* scores were used to calculate 95% CIs. Fisher r to z transformation revealed a significant difference in the correlations (Z=–2.97, *P*<.01). Additionally, intraclass correlation (2,1) estimates and their 95% CIs were calculated using SPSS (SPSS Inc), the absolute-agreement, single rater model indicates that the reliability of the app version of the questionnaire was similar to the paper version (Table 2).

**Table 2:** Intraclass correlations in SPSS using an absolute-agreement, single rater model.

	Intraclass correlation	95% CI	*F* test with a true value of 0			
			Value	*df*_1_	*df*_2_	Sig
App	0.842	0.812-0.868	11.689	416	416	0.000
Paper	0.810	0.783-0.834	9.526	721	721	0.000

The 3-factor structure of the 28-item CYRM-28, based on the model confirmed by Liebenberg and Unger [18], was estimated using a CFA with the Time 1 data set in AMOS 24. A maximum likelihood estimation CFA model was found to be parsimonious; however, the significant results on chi-square analysis indicate that the model did not adequately fit the data (*χ*^2^_15_=27.6 [n=541], *P*=.24). As large sample sizes can increase the likelihood of significant chi-square results, other indices of model fit are of particular interest. Table 3 includes a range of fit indices, all of which are within acceptable parameters.

**Table 3:** Model fit summary for the app version of CYRM-28^a^ confirmatory factor analysis.

	Chi-square (*df*)	*P* value	Goodness of fit	Comparative fit index	Root mean square error of approximation
Original model	43.8 (17)	>.01	0.94	0.98	0.54
Second model	27.59 (15)	.24	0.98	0.99	0.39

^a^CYRM-28: child and youth resilience measure.

Modification indices were examined, and several items were found to have significant shared error variance, including the following: relational (physical) and contextual (spiritual); individual (personal) and individual (peer). An exploration of the items included in each of these factors for multicollinearity between the items suggested that no item was so redundant with another item that it could be dropped (e1-e2, tolerance=1.00, variance inflation factor=1.00; e4-e8, tolerance=–1.00, variance inflation factor=1.00). As the shared error variance between all of these pairs of items was conceptually consistent with the domain assessed, a final model was respecified to free these correlated errors. This model was found to fit the data moderately well, and increased goodness of fit (*χ*^2^_15_=27.6 [n=541], *P*=.24); further details of fit can be seen in Table 2. The final confirmatory factor analytic model of the CYRM-28 indicated that the items were strongly correlated within factors rather than across factors, this replicates the findings from the original validity study [18]. Diagram 2 shows the error-covariances added to improve the model goodness of fit; each of these were low (*r*=0.12 and *r*=–0.15).

## Discussion

### Principal Findings

The aim of the study was to establish the adequacy of an app version of a previously validated paper version of a scale to measure resilience. The app and the paper versions of the scale presented the text of the items using Likert scales. The paper version allowed pupils to ask staff for support while in the app version, this was built into the device. The results indicate that the app had significantly better reliability in a test-retest analysis and had significantly higher internal consistency, as measured with the Cronbach α score. Scores across the demographic groups between the paper and app versions did not differ, indicating that the app version matches the paper version on the CYRM-28 when measuring resilience. Finally, the study supports the use of the CYRM-28 in a Scottish youth population [9-16].

### Comparison With Prior Work

Ungar [7] previously reported that resilience was not only a function of the individual, but also that environmental influences are important. The CFA reflected this understanding of resilience and further confirmed by Liebenberg and Ungar [17] earlier reported a 3-factor solution (individual, family relationships, and contextual). Furthermore, the CYRM-28 was designed to be used with the support of an adult professional (teacher or social worker) [18], and while this ensures that young people have understood the statements, it is not cost-effective and therefore is of use only to small groups of children who have been identified as vulnerable. Additionally, the pastoral system within Scottish schools is increasingly under strain. This study provides evidence that a sizable percentage of children would not seek support from their guidance teachers. The purpose of this study was to develop a low-cost scalable version of the questionnaire, which depends on an avatar to support understanding and encourages openness in adolescents. As discussed by Palmier-Claus [37], the app’s increased reliability, as evident from its high internal consistency, and in addition, participants were more likely to provide similar responses across time periods when using the app version. Previous studies indicated that the use of the avatar in the app would be a positive experience, and this has been replicated in this study. The students who completed the supplementary usability questions were generally positive about the avatar. It can be assumed that while app usage was time-limited, the participants were able to develop a relationship of trust with ALEX and were therefore open in their responses.

### Limitations

This study sought to explore how effective an avatar was in connecting with young people and collecting data about their home-lives and feelings. Our findings show that the app performed well at this level of data collection and a proof of concept has been met. However, for ethical reasons, it was decided to test this in a general population of young people, rather than adolescents who have been identified as vulnerable. Furthermore, while it can be argued that resilience is more observable among people who are facing trauma or difficult situations, the CYRM-28 has previously been used in general populations [17,19]. Nonetheless, further research that includes vulnerable participants would be warranted.

The final version of the app was designed to allow the participant as well as the professional to access information about the pupil. While it is important to develop highly reliable but easy-to-administer assessments, it is important that the results are of use to the teacher or social worker in helping support pupils. In this study, the reports were only available to guidance teachers and were for research purposes only. It is possible that knowledge of this had an impact on the participants’ answers. However, both groups (app and paper) were exposed to this variable. Furthermore, among the usability questions, pupils were asked about whether they had thought this knowledge had affected their answer, with the majority stating that it had not. Additionally, the app will be used in a setting in which reports will be available to experts such as teachers, educational psychologists, and social workers. It was important that this was incorporated in the trial. Parents had consented to reports being used in future studies about the usability of reports, and both groups of pupils were informed of this prior to the study as part of the assent process.

Current studies are exploring how professionals utilize feedback from an app, but another question not answered here is how the young people themselves react to instant feedback on an aspect of their psychological life. Additionally, a discussion on the use of the app within a broader health and social education setting should be developed. The authors strongly suggest that the app would be well-suited in ongoing curricula designed around assessing and developing aspects of well-being. Education practitioners and social workers should be involved in developing good practice in relation to the use of such apps. It is recommended that this forms part of a conversation between guidance teachers and young people, rather than the end result of an assessment. To that end, future research should consider how assessment apps can enable participants to communicate with their guidance teachers; this feature is of particular interest, given the findings of our study on the reluctance of pupils to approach their teachers.

### Conclusions

The app technology utilized in this study has shown strong reliability and validity in measuring resilience in young adult populations. Our findings demonstrate the efficacy of moving the CYRM-28 “gold-standard” measure of resilience to a web-based app-based platform. The benefits of avatar-led questioning in relation to young people’s understanding of resilience are evident; however, future studies should address how technology can be effectively integrated into existing practitioner-led support services within schools. 

## Abbreviations

CYRM-28: child and youth resilience measure
GIRFEC: getting it right for every child
SHANARRI: Safe, Healthy, Active, Nurtured, Achieving, Respected, Responsible, Independent.
